# Early Life Ambient Air Pollution and Child Neurodevelopment: Critical Review of Cohort Evidence

**DOI:** 10.3390/jox16060191

**Published:** 2026-10-09

**Authors:** Abdulrahman A. Al-Atram, Mohammad Elkossi, Shimaa Elwardany Aly, Ahmad Roshdy Ahmad, Mohamad-Hani Temsah, Walaa A. Afifi, Khaled Saad

**Affiliations:** 1 Psychiatry Division, Department of Medical Specialties, College of Medicine, Majmaah University, Majmaah 11952, Saudi Arabia; 2 Stepping Hill Hospital, Stockport SK2 7JE, UK; 3 Department of Primary Care Nursing, Faculty of Nursing, Al-Ahliyya Amman University, Amman 19328, Jordan; 4 Department of Pediatrics, College of Medicine, Jouf University, Sakaka 72388, Saudi Arabia; 5 Department of Pediatrics, College of Medicine, King Saud University, Riyadh 11461, Saudi Arabia; 6 Department of Mathematics and Statistics, College of Science in Yanbu, Taibah University, Yanbu Governorate 46422, Saudi Arabia; 7 Department of Pediatrics, Faculty of Medicine, Assiut University, Assiut 71516, Egypt

**Keywords:** ambient air pollution, particulate matter, nitrogen dioxide, neurodevelopment, child health, birth cohort, autism spectrum disorder, attention-deficit/hyperactivity disorder, environmental epidemiology

## Abstract

Background: Ambient air pollution is a pervasive exposure during pregnancy and childhood, when the central nervous system undergoes rapid development. Objective: To critically examine selected cohort evidence on prenatal and early-childhood exposure to PM_2.5_, PM_10_ and nitrogen dioxide (NO_2_), with particular attention to null findings, exposure assessment, confounding and causal interpretation. Methods: This narrative review used purposive evidence selection and targeted updating of PubMed-indexed records and publisher reports through 30 August 2026. Eligibility and selection priorities are stated explicitly. Representative studies were appraised qualitatively for exposure measurement, outcome ascertainment, confounding, selection, and multiplicity; no registered systematic review protocol, duplicate screening, formal risk-of-bias score, or de novo meta-analysis was used. Results: European and United States multicohort studies reported small associations with selected psychomotor, behavioural, and cognitive outcomes alongside substantial null findings. Canadian and South Korean cohorts further illustrate pollutant- and age-specific heterogeneity. Associations involving birth weight or prematurity require distinction between total and conditional effects. Placental, neuroimaging, and biomarker studies support biological plausibility but do not establish mediation or causation. A Chinese power-plant closure comparison provides complementary evidence, although concurrent temporal changes remain an alternative explanation. Conclusions: Early-life ambient air pollution may contribute to population-level neurodevelopmental risk, but the magnitude, pollutant specificity, and causal interpretation remain uncertain. The evidence does not establish pollution as a determinative cause of autism spectrum disorder (ASD), attention-deficit/hyperactivity disorder (ADHD), or an individual child’s developmental profile. More geographically inclusive cohorts and complementary causal designs are required.

## 1. Introduction

Brain development begins before birth and continues through childhood and adolescence. During fetal life and the first years after birth, neurogenesis, neuronal migration, synapse formation, axonal growth, myelination, and synaptic pruning occur in overlapping, developmentally time-dependent sequences. These processes can be influenced by maternal health, placental function, nutrition, infection, psychosocial adversity, toxicant exposure, and the postnatal environment. The timing of an exposure, therefore, requires consideration alongside its average concentration.

Ambient air pollution is a complex mixture rather than a single exposure. It includes particulate matter with an aerodynamic diameter of 2.5 μm or less (PM_2.5_), particulate matter with an aerodynamic diameter of 10 μm or less (PM_10_), ultrafine particles, nitrogen oxides, ozone, black carbon, polycyclic aromatic hydrocarbons, metals, and other combustion-related constituents. Composition varies by traffic density, industrial activity, power generation, residential fuel use, dust, agricultural activity, wildfires, meteorology, and season. PM_2.5_ mass and NO_2_ are therefore commonly interpreted as indicators of source mixtures or related emissions rather than as interchangeable measures of a single toxic agent.

The public health relevance is substantial. The 2021 World Health Organization annual air-quality guideline for PM_2.5_ is 5 μg/m^3^ [1]. Rentschler and Leonova estimated that 7.3 billion people are exposed to annual PM_2.5_ concentrations above this guideline and that approximately four-fifths of these individuals live in low- and middle-income countries [2]. Even a small causal shift in developmental test performance could have population importance because exposure is widespread; however, a population association does not predict the developmental trajectory of an individual child.

This critical narrative review examines whether prenatal and early-childhood ambient air pollution is associated with variation in cognitive, language, motor, and behavioural development, dimensional autistic or attention symptoms, clinically diagnosed ASD or ADHD, and intermediate neuroimaging phenotypes. Its contribution is an explicit comparison of adverse and null findings, accompanied by an appraisal of exposure error, environmental co-exposures, perinatal conditioning, and the limits of causal inference. This emphasis complements the broader mechanistic and harmonization reviews by Ha and Volk and colleagues [3,4]. The principal exposure metrics are PM_2.5_, PM_10_, and NO_2_; black carbon, particulate constituents, polycyclic aromatic hydrocarbons (PAHs), ozone, and indoor pollution are considered when they clarify source mixtures or interpretation. Europe and North America supply most of the multicohort evidence; selected Asian reports broaden the discussion without implying global representativeness.

## 2. Materials and Methods

### 2.1. Review Design and Evidence Selection

This is a critical narrative review using purposive, report-level evidence selection. The principal evidence comprises human birth or childhood cohorts and harmonized multicohort analyses that assigned ambient exposure during pregnancy or approximately the first five years of life and assessed subsequent neurodevelopment using standardized tests, symptom instruments, clinical diagnoses, or neuroimaging. Eligible reports needed an identifiable population, exposure window and method, outcome definition, and interpretable adjusted findings. Systematic reviews, methodological studies, selected family-based case–control studies, and an emission-reduction cohort comparison were used for context rather than combined quantitatively with the main cohorts. Animal experiments were considered only for mechanistic plausibility.

Targeted bibliographic verification and updating were completed on 30 August 2026 using online searches of PubMed-indexed records, journal publisher websites, PubMed Central, and author repositories. Core query combinations were “air pollution prenatal child neurodevelopment cohort”, “air pollution early childhood autism ADHD cohort”, and “air pollution child neurodevelopment natural experiment sibling mediation”; supplementary searches used study titles, author names, pollutant constituents, indoor exposure, exposure-measurement error, and mixture methods. No lower publication-date limit was applied; the update ended on 30 August 2026. English-language reports or records were used, and no comprehensive multilingual search was undertaken. Adult-only studies, studies without a relevant developmental outcome or identifiable early-life ambient exposure, conference abstracts without a verifiable report, and duplicate descriptions of the same analysis were not used as primary evidence.

### 2.2. Framework for Appraisal

Selection prioritized harmonized multicohort analyses, large cohorts with clinically ascertained disorders, prospective cohorts with repeated developmental assessments, and studies illustrating exposure or causal-methodological issues. Findings were retained irrespective of statistical significance. ESCAPE, and the European attention analysis include constituent cohorts such as INMA, Generation R, the Amsterdam Born Children and their Development study, and the Swedish Child and Adolescent Twin Study; these populations are therefore not wholly absent. Canadian, South Korean, and Chinese studies were added to illustrate different settings and designs. Overlapping cohorts were identified and were not treated as independent replications. This selection logic improves transparency but cannot eliminate narrative-review selection bias or establish exhaustive coverage.

For each representative report, the appraisal considered exposure measurement, outcome validity, confounder control, selection or attrition, conditioning on perinatal variables, and multiple testing. Table 1 records reported strengths and vulnerabilities rather than an aggregate quality score. No independent duplicate screening, adjudication, or duplicate appraisal was undertaken, and no standardized risk-of-bias instrument was applied. Details not ascertainable from the consulted report or indexed abstract are identified explicitly and were not inferred. This review is neither preregistered nor PRISMA-compliant and does not claim a complete study census. Effect estimates retain the original exposure increments and outcome scales, which preclude a common pooled magnitude.

Outcome constructs were kept distinct. Parent- or teacher-rated attention symptoms, Child Behavior Checklist scores, and executive-function tasks were not equated with a clinical ADHD diagnosis. Autistic-trait measures were similarly distinguished from clinically ascertained ASD. Mechanistic and neuroimaging findings were interpreted as possible intermediate phenotypes rather than proof of a clinical causal pathway.

## 3. Multicohort Evidence and Exposure Assessment

### 3.1. Value and Limits of Multicohort Studies

Multicohort studies can assess whether a finding is reproduced across populations with different pollutant mixtures, residential patterns, social conditions, and health-care systems. The European Study of Cohorts for Air Pollution Effects (ESCAPE) developed standardized NO_2_ and NO_X_ land-use regression models in 36 European study areas [5]. The United States ECHO-PATHWAYS studies applied common exposure estimates and analytic protocols across three pregnancy cohorts. Harmonization improves comparability but does not eliminate residual confounding, exposure error, or outcome heterogeneity.

### 3.2. Exposure Assignment and Co-Exposure

Most cohorts estimate maternal or child exposure at the residential address using land-use regression, satellite-derived products, chemical transport models, or hybrid spatiotemporal models. These approaches improve geographic and temporal resolution compared with assignment from a fixed monitor alone. They remain proxy measures for personal dose. They usually do not capture time spent at school, childcare, work, or in transit; indoor infiltration; cooking and heating sources; tobacco smoke; occupational exposure; filtration; or residential mobility.

Pollutants, traffic noise, heat, green space, and neighborhood deprivation commonly co-occur. A single-pollutant coefficient may represent a source mixture or correlated social and environmental determinants. Multipollutant models can be unstable when exposures are strongly collinear. Therefore, pollutant-specific coefficients should not automatically be interpreted as evidence of an independent toxic effect of that pollutant.

Indoor exposure is a material part of personal dose. Outdoor infiltration depends on ventilation, building characteristics, and filtration, while cooking, heating, and tobacco smoke introduce additional sources. Tokuda and colleagues combined modelled outdoor exposure with one-week indoor measurements at child ages 1.5 and 3 years in a Japanese birth cohort; they reported different associations of outdoor and indoor pollutants with behaviour and cognition at age six [6]. Short indoor sampling periods may not characterize long-term dose. The present review principally concerns ambient exposure and does not estimate the separate effects of household solid-fuel smoke or personal indoor mixtures.

### 3.3. Particle Composition and Exposure Measurement

**Figure 1:**
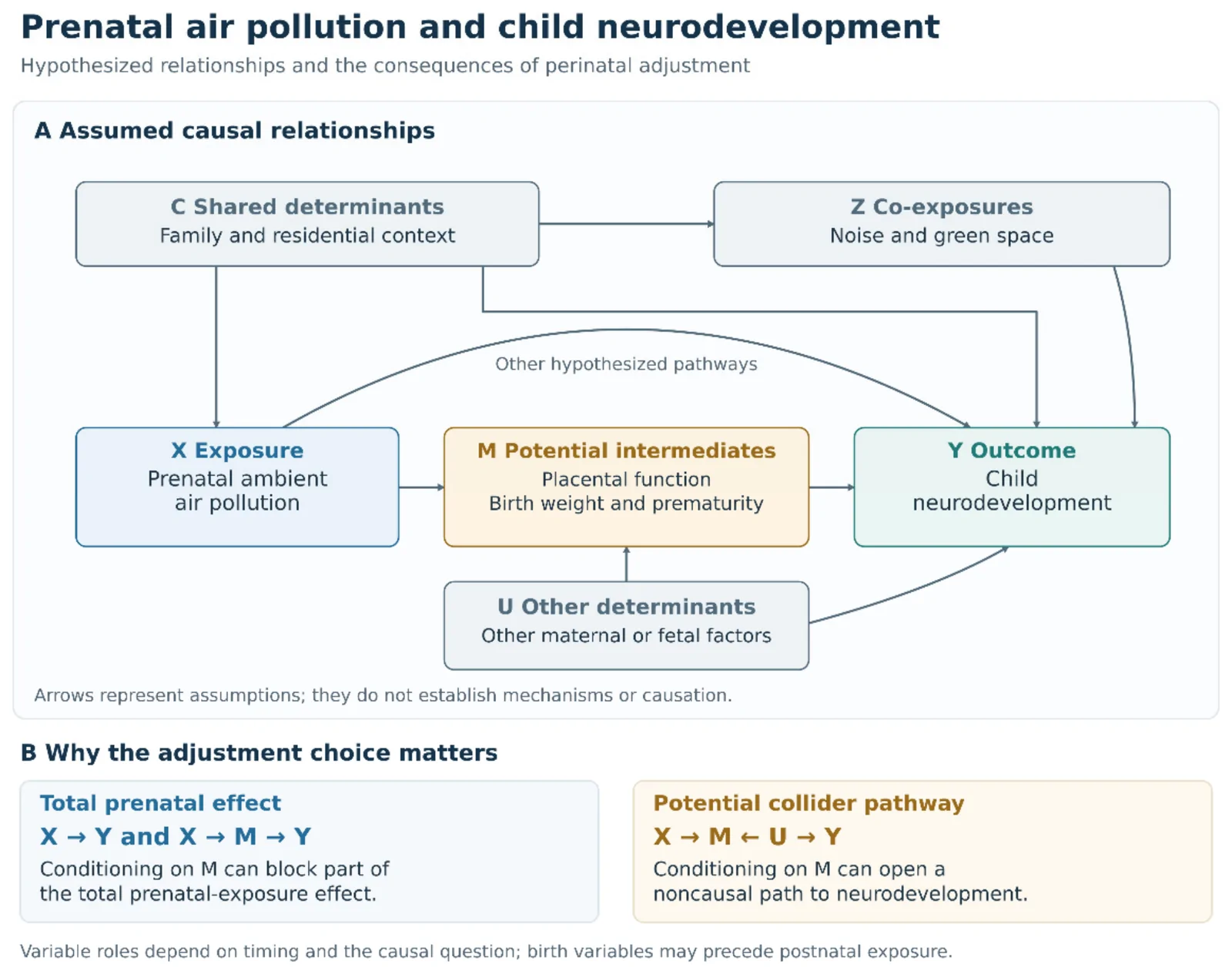
Hypothesized causal framework for prenatal air pollution and child neurodevelopment. (**A**) Shared family and residential determinants (C) may influence prenatal air-pollution exposure (X), environmental co-exposures (Z; noise and green space), and child neurodevelopment (Y). Placental function, birth weight, and prematurity (M) are represented as potential intermediates, while other maternal or fetal factors (U) may affect both M and Y. (**B**) Conditioning on M may block part of the total prenatal-exposure effect and open a noncausal pathway through U. Arrows indicate hypothesized relationships rather than established mechanisms or causation. Variable roles depend on exposure timing and the causal question; birth characteristics may precede postnatal exposure.

Equal PM_2.5_ mass does not imply equal source composition or toxicological potential. Ultrafine-particle number, particle surface area, black carbon, transition metals, and PAHs may capture different combustion or non-exhaust sources. Lubczyńska and colleagues evaluated eight elemental constituents in 7246 children from four European cohorts and reported an association of airborne iron with fine-motor function [7]. This is component-specific epidemiological evidence, but it does not establish that one constituent is uniquely responsible or provide a validated ranking of neurotoxicity. Correlated constituents, geographically varying mixtures, and exposure-model error remain important. Source-apportionment data should therefore complement mass-based metrics. Black carbon and PM_2.5_ absorbance are useful combustion indicators, but neither represents the complete particle mixture. Ozone differs in formation, spatial distribution, and seasonality from traffic-related NO_2_; it was included in the later ECHO-PATHWAYS executive-function analysis [8]. Ultrafine particles are poorly represented by routine PM mass monitoring. These metrics are discussed selectively because consistent early-life exposure reconstruction and directly comparable cohort outcomes are less available; the review does not claim comprehensive coverage of every gaseous or particulate pollutant. Figure 1 provides a conceptual causal framework for separating exposure and shared residential determinants from potential prenatal intermediates.

### 3.4. Representative Cohort Evidence

**Figure 2:**
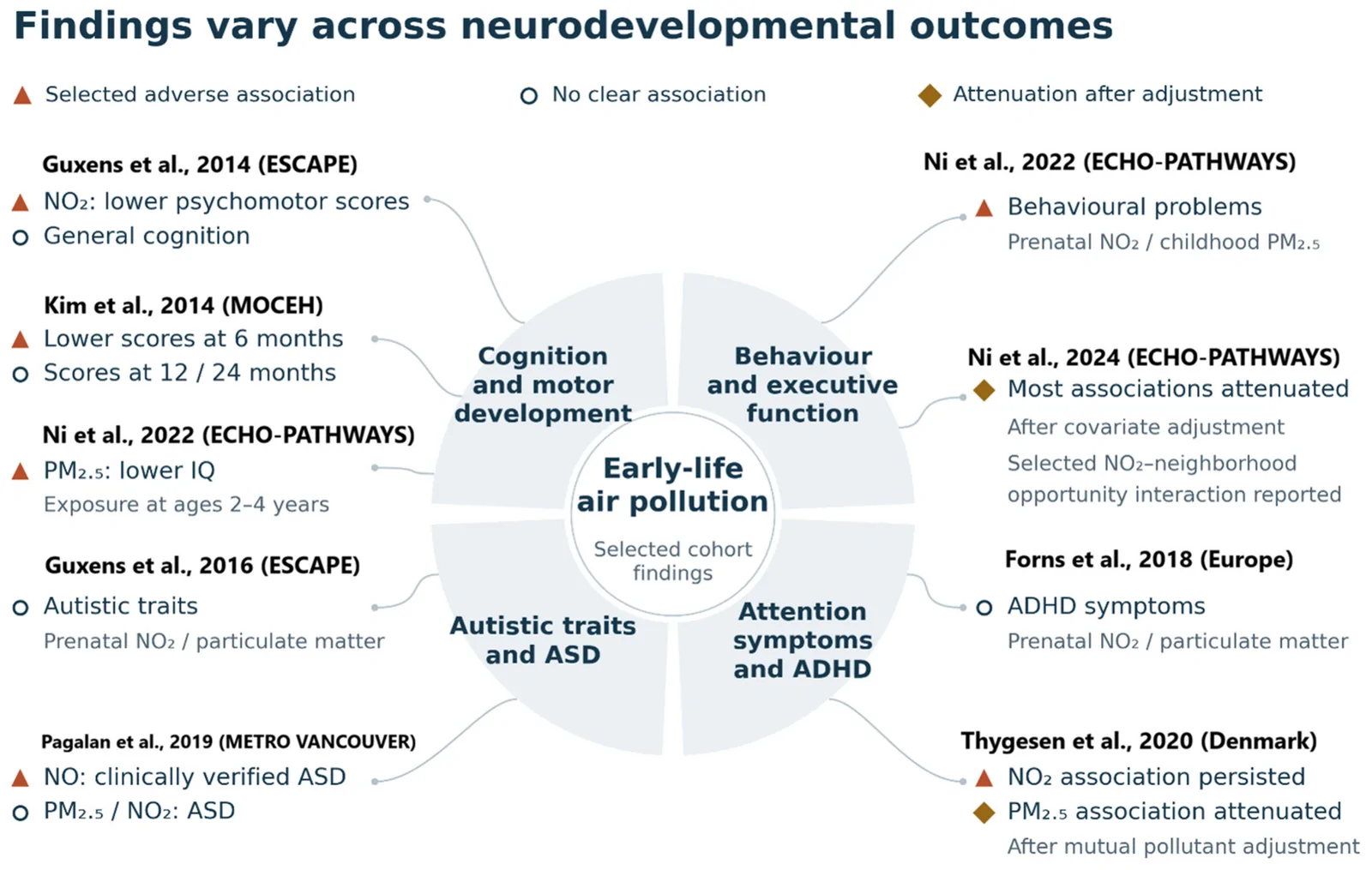
Selected cohort findings on early-life air pollution and child neurodevelopment across four domains: cognition and motor development; behaviour and executive function; autistic traits and autism spectrum disorder (ASD); and attention symptoms and attention-deficit/hyperactivity disorder (ADHD). Triangles mark selected adverse associations, open circles show no clear association, and diamonds indicate attenuation after covariate or mutual pollutant adjustment. Patterns vary within and across studies by pollutant, developmental age, and outcome. The map is descriptive and does not provide comparable effect sizes, pooled estimates, evidence grades, or causal inference [8,9,10,11,12,16,18,19]. Study details and estimates are provided in Table 1.

Table 1 summarizes representative studies [8,9,10,11,12,13,14,15,16,17,18,19,20], including their exposure methods, reported adjustment variables, and main interpretive limitations. Absence of explicit noise or green-space adjustment is distinguished from adjustment for broader socioeconomic indicators. Figure 2 compares selected adverse, null, and attenuated findings across major multicohort and population-based studies, grouped by neurodevelopmental domain. The comparison is descriptive and does not pool effect estimates or count independent replications.

**Table 1:** Representative early-life air-pollution studies and report-level critical appraisal.

Study and Population	Outcome and Age	Exposure Window and Method	Principal Adjusted Findings	Reported Adjustment Variables	Environmental Context	Strengths and Limitations
Guxens 2014 [9] Europe; *n* = 9482	Cognitive and psychomotor development; ages 1–6 years	Prenatal NO_2_, NO_x_, and PM; residential ESCAPE land-use regression (LUR), temporally adjusted	NO_2_: psychomotor difference −0.68 points per 10 μg/m^3^ (95% CI −1.25 to −0.11); general cognitive findings null	Adjusted cohort estimates; individual covariate list not available in the consulted indexed abstract	Noise/green-space terms NR	Large harmonized sample; proxy exposure, between-cohort outcome differences and incomplete accessible adjustment detail
Guxens 2016 [10] Four European cohorts; *n* = 8079	Autistic traits; ages 4–10 years	Prenatal NO_2_ and PM; temporally adjusted residential LUR	No association with prenatal NO_2_ or PM; trait outcomes should not be equated with diagnosed ASD	Maternal education, origin, age, BMI, height, smoking and parity; child sex/age, season, urbanicity and evaluator	Urbanicity; no explicit noise/green-space term in main model	Harmonized multicohort analysis; symptom ascertainment and residual contextual confounding
Forns 2018 [11] Eight European cohorts; *n* = 29,127	Parent/teacher ADHD symptoms; ages 3–10 years	Prenatal PM and NO_2_; temporally adjusted residential LUR	Null ADHD-symptom findings; NO_2_ OR 0.95 per 10 μg/m^3^ (95% CI 0.89–1.01)	Maternal education/SES, origin, age, BMI, height, smoking and parity; child sex/age, season, urbanicity and evaluator	Urbanicity; no explicit noise/green-space term in main model	Large sample and missing-data/attrition analyses; heterogeneous questionnaires and residual co-exposure confounding
Ni 2022 [12] ECHO-PATHWAYS; *n* = 1967	CBCL Total Problems and full-scale IQ; ages 4–6 years	Prenatal and ages 0–2/2–4 years; PM_2.5_/NO_2_; biweekly spatiotemporal models with address history	Selected prenatal NO_2_ and postnatal PM_2.5_ associations; IQ −2.63 per 2 μg/m^3^ at ages 2–4 (95% CI −5.08 to −0.17)	Income, education, maternal IQ/depression, smoking/alcohol, household factors, child factors, and neighborhood opportunity	Educational/economic COI; no explicit noise/green-space term	Detailed adjustment; selected cohorts, residential-dose error, multiplicity and windows not consistently confirmed by lag models
Ni 2024 [8] ECHO-PATHWAYS; *n* = 1235	Flexibility, working memory and inhibition; school age	Birth–4 years; PM_2.5_, NO_2_ and ozone; biweekly spatiotemporal models with address history	Most crude inverse findings attenuated after adjustment; selected NO_2_–neighborhood opportunity interaction	Income, education, maternal mental health/behaviour, child factors, and COI; the extended model included birth characteristics	Socioeconomic/educational COI; no explicit noise/green-space term	Covariate sensitivity supports caution; attrition, exposure error, correlated outcomes and multiple interaction tests
Perera 2024 [13] New York City; *n* = 470	Bayley-II mental and psychomotor indices; ages 1–3 years	Prenatal PM_2.5_/NO_2_; daily residential spatiotemporal models; trimester and lag analyses	Selected mental-development associations at ages 1 and 3; no psychomotor association	Maternal education, IQ, demoralization and tobacco smoke; ethnicity/sex; HOME score additionally at age 3	Home-environment score at age 3; no explicit noise/green-space term	Prospective repeated testing and multiplicity correction; selected nonsmoking cohort and differential analytic inclusion
Morgan 2023 [14] Southern California; *n* = 161	Bayley-III; age 2 years	Prenatal PM_2.5_, PM_10_ and NO_2_; residential estimates from monitoring data; distributed-lag models	Lower cognitive scores in selected models; only PM_10_ composite/scaled motor findings survived 10% FDR correction	SES, breastfeeding, maternal pre-pregnancy BMI, child sex, birth weight, and gestational timing	SES; no explicit noise/green-space term	Small selected sample, correlated tests and perinatal conditioning; maternal IQ/home measures unavailable
Bonthrone 2026 [15] London dHCP; *n* = 498; 125 preterm	Bayley-III; ages 17.3–34.5 months	Gestational PM_2.5_/PM_10_/NO_2_; London Air Pollution Toolkit dispersion/measurement models; maternal postcode	First-trimester language associations; gestational motor associations in the preterm subgroup	Sex, ethnicity, pregnancy complications, deprivation, birth-weight z-score, gestational age/home language, or respiratory support by model	Neighborhood deprivation; no explicit noise/green-space term	Clinical characterization; perinatal conditioning/stratification, postcode exposure, selection, and unmeasured postnatal exposure
Thygesen 2020 [16] Denmark; *n* = 809,654	Clinically recorded ADHD; follow-up after age 5	Cumulative NO_2_/PM_2.5_ in first 5 years; residential dispersion modelling	NO_2_ association persisted; PM_2.5_ substantially attenuated in two-pollutant models	Age, calendar year, sex, parental education, and income	Parental SES; no explicit noise/green-space term in reported model	Large registry and longitudinal addresses; diagnosis/access differences, familial confounding and co-emitted pollutants
Lewandowska 2025 [17] Poland; *n* = 425; 116 ADHD	Diffusion MRI; ages 10–13 years	Prenatal and ages 0–4 years; NO_2_/PM_10_; residential hybrid LUR	Selected global NO_2_ diffusion associations; fixel analyses null; no differential association by ADHD status	Age, sex and ADHD status; parental education in sensitivity analysis	Education sensitivity analysis; no explicit noise/green-space term	Objective imaging with correction; imaging selection, limited main-model SES control and intermediate outcomes
Kim 2014 [18] South Korea MOCEH; *n* = 520	Korean Bayley-II; ages 6, 12, and 24 months	Prenatal PM_10_/NO_2_; inverse-distance weighting of monitoring data	Lower mental/psychomotor indices at 6 months; associations not significant at 12 or 24 months	Multivariable models; individual covariate list not available in the consulted indexed abstract	Noise/green-space terms NR	Repeated prospective assessment; age-specific results, monitoring-based exposure and incomplete accessible adjustment detail
Pagalan 2019 [19] Canada; 132,256 births; 1307 ASD	Clinically verified ASD by age 5	Prenatal PM_2.5_, NO and NO_2_; temporally adjusted high-resolution LUR	Adjusted PM_2.5_ and NO_2_ intervals included null; NO OR 1.07 per 10.7 ppb (95% CI 1.01–1.13)	Child sex, birth month/year, maternal age/origin, neighborhood income and urbanicity	Neighborhood income/urbanicity; no explicit noise/green-space term	Large population cohort and clinical diagnosis; incomplete familial/lifestyle adjustment and correlated trimester exposures
Tang 2014 [20] China Tongliang; 150 before and 158 after closure	Gesell developmental quotients; age 2	Coal-plant closure cohort comparison; cord PAH–DNA adducts and BDNF; 269 with both biomarkers	Postclosure adducts lower and BDNF higher; associations with selected developmental domains	Cord lead/mercury, tobacco smoke, maternal age/education, gestational age, and child sex	Maternal education/tobacco; no explicit noise/green-space term	Source-reduction comparison and biomarkers; no concurrent control, temporal confounding, and smaller outcome follow-up samples

Notes: Sample sizes refer to the reported cohort or analytic sample; outcome-specific numbers may differ. Adjustment lists are abbreviated and are not formal risk-of-bias scores. NR denotes detail not reported or not ascertainable in the consulted source and does not demonstrate that a variable was omitted from the original analysis. Socioeconomic proxies are distinguished from direct noise or green-space measures. Perinatal adjustment must be interpreted in relation to the exposure window and estimand. ECHO analyses overlap in cohort membership. Abbreviations: ASD, autism spectrum disorder; ADHD, attention-deficit/hyperactivity disorder; BDNF, brain-derived neurotrophic factor; BMI, body mass index; CBCL, Child Behavior Checklist; CI, confidence interval; COI, Child Opportunity Index; dHCP, Developing Human Connectome Project; ECHO, Environmental influences on Child Health Outcomes; ESCAPE, European Study of Cohorts for Air Pollution Effects; FDR, false discovery rate; HOME, Home Observation for Measurement of the Environment; IQ, intelligence quotient; LUR, land-use regression; MRI, magnetic resonance imaging; OR, odds ratio; PAH, polycyclic aromatic hydrocarbon; PM, particulate matter; SES, socioeconomic status.

## 4. Cognitive Language and Motor Development

### 4.1. Harmonized European Evidence

The 2014 ESCAPE analysis of six European birth cohorts included 9482 children assessed between 1 and 6 years of age. Prenatal NO_2_ was associated with a 0.68-point lower global psychomotor development score per 10 μg/m^3^ increase (95% CI −1.25 to −0.11); no association was reported between the examined pollutants and general cognitive development [9]. This pattern is important because it does not support a uniform effect across developmental constructs. It supports a small pooled psychomotor difference in this dataset, not a universal cognitive impairment caused by NO_2_.

### 4.2. Prospective Cohorts and Developmental Timing

The New York City cohort studied by Perera and colleagues reported inverse associations between selected prenatal PM_2.5_ and NO_2_ windows and Bayley-II cognitive scores at ages 1 and 3 years, but no significant association with the Psychomotor Development Index [13]. Morgan and colleagues reported associations of prenatal PM_2.5_ and PM_10_ with lower Bayley-III cognitive scores at age 2 years in 161 Southern California children [14]. However, after adjustment for multiple testing, only the PM_10_ associations with composite and scaled motor scores remained significant at the study’s 10% false-discovery-rate threshold. The cognitive findings, therefore, require particular caution. Multiple correlated exposure periods and outcomes were evaluated; highlighted intervals are hypotheses about susceptibility rather than established biological boundaries.

Bonthrone and colleagues studied 498 toddlers in the Developing Human Connectome Project, including 125 born preterm. Higher first-trimester PM_2.5_, PM_10_, and NO_2_ exposure was associated with lower language scores, and gestational exposure was associated with lower motor scores in the preterm subgroup [15]. The models included gestational age and/or birth-weight z-score, together with socioeconomic deprivation and clinical covariates. These are conditional associations; perinatal adjustment and stratification require caution when interpreting a total prenatal-exposure effect. Replication is needed. A systematic review of preschool- and school-aged children likewise described heterogeneous evidence across developmental domains [21].

The South Korean MOCEH study followed 520 mother–child pairs assessed repeatedly using the Korean Bayley Scales. Prenatal PM_10_ and NO_2_ were associated with lower mental and psychomotor indices at six months, but associations were not significant at 12 or 24 months [18]. This age-dependent pattern cautions against equating an early test-score association with persistent impairment. It also broadens the evidence beyond European and North American populations.

Across cognitive, language, and motor domains, the evidence is not uniform by pollutant, age, exposure period, or assessment instrument. Small group-level differences on standardized tests are not equivalent to a diagnosis, a functional impairment, or a need for clinical intervention in an individual child.

## 5. Behavioural Outcomes: Autism Spectrum Disorder and Attention Deficit Hyperactivity Disorder

### 5.1. Behavioural Problems and Executive Function

Ni and colleagues analysed 1967 mother–child dyads from three United States pregnancy cohorts. Higher prenatal NO_2_ averaged across pregnancy was associated with a 1.24-point higher Child Behavior Checklist Total Problems score per 2 ppb (95% CI 0.39 to 2.08). PM_2.5_ exposure at ages 2–4 years was associated with a 3.59-point higher Total Problems score (95% CI 0.35 to 6.84) and a 2.63-point lower IQ (95% CI −5.08 to −0.17) per 2 μg/m^3^ [12]. The IQ interval is compatible with an average decrement ranging from very small to approximately five points; its upper limit is close to zero. Conventional statistical significance, therefore, does not establish a precise magnitude, clinically meaningful impairment, or causation. These dimensional measures are not psychiatric diagnoses.

The subsequent ECHO-PATHWAYS executive-function analysis included 1235 children and assessed cognitive flexibility, working memory, and inhibitory control. Most inverse associations present in crude models did not persist after adjustment. An interaction between NO_2_ and neighborhood educational opportunity was reported for working memory, but no interaction was found by child sex or maternal education [8]. This finding is a material counterweight to a general claim that United States multicohort evidence consistently demonstrates pollution-related executive dysfunction.

### 5.2. Autism Spectrum Disorder and Autistic Traits

The ESCAPE analysis of four European cohorts included 8079 children with autistic-trait assessments at 4–10 years and found no association between prenatal NO_2_ or particulate matter and autistic traits [10]. A dimensional trait scale is not interchangeable with clinician-diagnosed ASD, but this large null multicohort result remains important when interpreting the broader literature.

In Metro Vancouver, Pagalan and colleagues studied 132,256 births, with 1307 ASD diagnoses by age five. In adjusted models, prenatal PM_2.5_ and NO_2_ associations were compatible with the null, whereas nitric oxide was associated with a modest increase in ASD odds [19]. High-resolution exposure models and clinically verified ascertainment were strengths, but incomplete familial and environmental covariate information remained relevant. These findings demonstrate pollutant-specific heterogeneity within a single population rather than a uniform association across traffic-related metrics.

Lin and colleagues reported pooled relative risks of 1.31 (95% CI 1.08 to 1.58) for prenatal PM_2.5_ and 1.64 (95% CI 1.16 to 2.34) for early-childhood PM_2.5_ per 10 μg/m^3^ in an exposure–response meta-analysis of 11 studies [22]. The evidence included two cohort and nine case–control studies, so the pooled estimates do not represent a purely prospective-cohort evidence base. Differences in ASD ascertainment, exposure modelling, and adjustment limit interpretation. Lam and colleagues also reported selected pooled associations, with methodological limitations and between-study heterogeneity [23]. Neither synthesis establishes pollutant-specific causation.

ASD ascertainment may be influenced by access to specialist assessment, referral patterns, diagnostic practice, and parental recognition of developmental differences. Neighborhood socioeconomic conditions can affect both exposure and diagnostic ascertainment. These features may leave residual confounding even after conventional covariate adjustment, and they are central to causal interpretation [24].

### 5.3. Attention Deficit Hyperactivity Disorder and Attention-Related Outcomes

Forns and colleagues analysed 29,127 mother–child pairs from eight European cohorts and found no evidence of increased attention-deficit/hyperactivity symptoms at ages 3–10 years with prenatal PM or NO_2_ exposure [11]. In contrast, the Danish nationwide cohort of 809,654 children reported positive single-pollutant associations of exposure during the first five years of life with clinically recorded ADHD. After mutual pollutant adjustment, the NO_2_ association persisted, whereas the PM_2.5_ association was substantially attenuated [16]. These studies differ in exposure timing and outcome ascertainment; their findings cannot be treated as direct contradictions or pooled on a single scale.

A systematic review restricted to clinically diagnosed ADHD found heterogeneous evidence and did not pool results because diagnostic codes, exposure windows, and covariate sets varied materially across studies [25]. A 2026 meta-analysis reported positive associations for PM_2.5_, PM_10_, and NO_2_ in continuous-exposure models, but the reported exposure increments differed across source studies, and heterogeneity was very high for several analyses [26]. These syntheses support further investigation, but they do not resolve pollutant specificity or causality.

Clinical ADHD diagnoses, rating-scale symptoms, and executive-function measures should remain conceptually separate. ADHD is diagnosed by a comprehensive assessment of symptoms, impairment, developmental history, and alternative explanations. A higher mean attention-problem score does not establish an increase in clinically diagnosed ADHD.

## 6. Neuroimaging and Biological Plausibility

### 6.1. Neuroimaging Findings

Neuroimaging can identify intermediate brain phenotypes, but it does not by itself establish a causal pathway to clinical outcomes. Peterson and colleagues reported associations of prenatal air-pollution exposure with altered brain structure, functional activation, and metabolism in childhood [27]. In the NeuroSmog diffusion magnetic resonance imaging study, early-life NO_2_ was associated with lower global fractional anisotropy and higher mean diffusivity. Fixel-based analyses were null, and ADHD diagnosis did not modify the association [17].

Imaging studies are vulnerable to selection and technical bias. Children who complete magnetic resonance imaging may differ from the full cohort in health, socioeconomic conditions, anxiety, attention, and access to study visits. Head motion, image quality, analytic choices, and multiple comparison correction can also influence results. Larger harmonized imaging cohorts are needed before imaging findings are considered evidence of a settled causal mechanism.

### 6.2. Proposed Biological Pathways

An integrated mechanistic model distinguishes maternal exposure during pregnancy from the child’s own inhalational exposure after birth. Maternal pulmonary oxidative stress and inflammation may affect systemic vascular function, placental perfusion, and fetal oxygen or nutrient supply, with downstream effects on neuronal maturation. After birth, systemic inflammatory signalling may alter blood–brain barrier function and glial responses. Experimental vehicle-emission exposure altered blood–brain barrier permeability in mice [28]; labelled ultrafine particles reached the olfactory bulb in rats [29]. These experiments support possible pathways but do not quantify their contribution at typical human exposures. Olfactory transport concerns the inhaling individual and should not be assumed to be a route of prenatal fetal exposure. Pulmonary sensory-afferent and vagal signalling is another proposed neuroimmune route, but its contribution to human developmental outcomes remains unresolved [3,4]. Bové and colleagues detected black carbon within placental tissue, including the fetal side, in a selected sample of 20 term placentas and five placentas from pregnancies ending earlier [30]. Optical sectioning and signal-characterization experiments addressed external contamination. Residential black-carbon exposure was positively associated with placental particle burden; the study therefore reported an exposure–burden relationship. Its small, selected sample, proxy exposure estimates, and lack of developmental follow-up limit interpretation. Independent replication with procedural blanks and complementary particle-identification methods remains valuable. Fetal-side placental deposition does not establish delivery to the fetal brain, a developmental dose–response relationship, or neurotoxicity. Human biomarker findings do not consistently demonstrate mediation. In the Tongliang cohorts, Tang and colleagues observed lower PAH–DNA adducts and higher cord-blood brain-derived neurotrophic factor (BDNF) after a coal-fired power plant closed; adducts and BDNF were associated with selected developmental-quotient domains [20]. These associations support a mechanistic hypothesis but do not alone establish a causal indirect effect. In a subsequent primary analysis, Lee and colleagues found that LINE-1 methylation related to prenatal PAH biomarkers and later IQ, but the formal mediation analysis did not establish LINE-1 as a mediator of the exposure–IQ association [31]. Cord-blood methylation is not a direct measure of brain epigenetic regulation. These findings illustrate why biomarker associations and demonstrated mediation must be distinguished.

## 7. Developmental Timing Susceptibility and Equity

### 7.1. Timing and Effect Modification

The cohort literature does not identify a universal susceptible gestational window. Adjacent exposure periods are correlated, and apparently, trimester-specific associations may reflect collinearity, imprecise pregnancy dating, multiplicity, pollutant composition, or outcome age. Gestational-week analyses should be anchored to a common biological origin, usually estimated conception, and should report how gestational duration and delivery-related lags are handled. A lag counted backward from delivery does not correspond to the same gestational week in every pregnancy. Distributed-lag analyses require prespecified smoothing, joint modelling of correlated periods, an appropriate multiple-testing strategy, and replication of both the cumulative association and any proposed window. Ni and colleagues’ distributed-lag analyses did not confirm most highlighted windows [12]. Potential modifiers include preterm birth, fetal growth, child sex, maternal nutrition, psychosocial stress, parental education, neighborhood conditions, genetic susceptibility, and co-exposure to tobacco smoke or metals. Results by child sex are inconsistent. Preterm birth warrants further study because it is associated with a higher baseline risk of adverse developmental outcomes, but a single observed interaction does not establish a clinical risk category.

### 7.2. Global and Social Inequities

The mismatch between the global exposure burden and cohort infrastructure is a key limitation. Most well-characterized neurodevelopmental cohorts are based in Europe and North America, whereas a large share of individuals living above the World Health Organization PM_2.5_ guideline reside in low- and middle-income countries [2]. Findings from lower-exposure settings should not be assumed to quantify risk in populations with higher concentrations, household combustion exposure, or different source mixtures.

Within countries, traffic-related pollution may co-occur with noise, poorer housing quality, crowding, tobacco smoke, fewer opportunities for residential mobility, and social disadvantage. These conditions can independently affect child development. Cohorts in South Asia, sub-Saharan Africa, Latin America, and other highly exposed regions should include household and personal exposure data, repeated residential histories, nutritional assessments, and developmental instruments validated in local languages and cultural contexts.

## 8. Methodological Challenges and Causal Inference

### 8.1. Exposure Error and Confounding

Exposure misclassification remains a persistent concern. In a simple linear model with independent classical error, random error in measured exposure generally attenuates the coefficient. Under ideal independent Berkson error, actual exposures vary around assigned group predictions, principally reducing precision without the same linear attenuation. Residential predictions can contain both types, as well as spatially correlated and heterogeneous error; nonlinear models, co-pollutant adjustment, and exposure-dependent error violate these simple expectations [32]. Consequently, a null association may reflect imprecision or exposure error, while a positive association may also be distorted. Validation subsamples, personal and indoor monitoring, residential histories, and propagation of prediction uncertainty are preferable to assuming that all error is conservative. Confounding by social and environmental context is an important alternative explanation for many findings. Traffic-related pollution is associated with noise, road safety, housing, green space, neighborhood deprivation, stress, and access to health care. Maternal smoking, alcohol exposure, nutritional status, maternal mental health, parental neurodevelopmental traits, and family socioeconomic circumstances may influence both residential location and child outcomes. Adjustment for measured covariates reduces but does not eliminate this concern.

Contextual adjustment is uneven. Ni and colleagues included household income, maternal education, smoking, maternal psychological measures, and educational/economic Child Opportunity Index domains [8,12]. Bonthrone and colleagues included neighborhood deprivation, whereas the Canadian ASD cohort used neighborhood income and urbanicity [15,19]. These variables do not directly measure traffic noise, green space, indoor sources, or every aspect of street-level deprivation. The absence of explicit noise or green-space terms in reported main models leaves co-exposure confounding possible; it does not establish its direction or magnitude. Uniform residential buffers and repeated contextual measurements would improve comparisons.

### 8.2. Perinatal Conditioning and Mediation

Covariate adjustment requires an explicit causal estimand and exposure window [33]. Figure 1 distinguishes shared determinants and environmental co-exposures from possible placental and perinatal mediators. For a total prenatal-exposure effect, routine adjustment for birth weight or prematurity can block an indirect pathway. If pollution and other maternal or fetal factors both influence a perinatal variable, conditioning on that common effect can open a noncausal path to neurodevelopment. Conversely, gestational age at birth can precede postnatal exposure and may then serve a different causal role. Mediation analysis requires temporally ordered measurements, control of exposure–mediator and mediator–outcome confounding, and appropriate handling of mediator–outcome confounders affected by exposure. A regression coefficient adjusted for a mediator is not automatically an identifiable direct effect. The presence of a perinatal adjustment term is not proof of bias, and this narrative review cannot provide a defensible proportion of studies that are biased by mediator conditioning. Morgan and Bonthrone explicitly adjusted prenatal-exposure analyses for birth weight and/or gestational age [14,15]. Their estimates should be interpreted in relation to those conditioning decisions. Ni’s postnatal executive-function analysis also considered birth characteristics, whose role differs because they preceded childhood exposure [8]. Study-specific diagrams and comparisons of total-effect and conditional models are needed to evaluate the consequences rather than assign a percentage from covariate lists alone. Outcome heterogeneity, selective attrition, and multiple testing further limit comparability. Studies may examine several pollutants, exposure windows, and developmental measures, increasing the chance of isolated statistically significant results. Conclusions should rely on convergence across outcomes and study designs, including well-described null findings, rather than on a single association.

### 8.3. Family-Based Comparisons and Emission Reduction Studies

Family-based designs can reduce shared familial confounding but require careful definition. Ghahari and colleagues used related family controls in an Iranian case–control study [34]; this is not equivalent to a prospective sibling fixed-effects analysis. Related controls do not remove pregnancy-specific smoking, stress, residential changes, ascertainment differences, or correlated exposure error. True sibling comparisons also lose precision when pollution varies little between pregnancies and can amplify exposure-measurement error. These limitations explain why family-based evidence must be interpreted alongside conventional cohorts rather than treated as a definitive causal test.

The Tongliang power-plant closure provides a concrete emission-reduction comparison: 150 mother–newborn pairs were enrolled before closure and 158 afterwards [20]. Lower PAH biomarkers and favorable changes in BDNF and selected developmental measures after closure strengthen the rationale for source-reduction studies. However, this nonrandomized comparison lacked a concurrent unexposed control population and remains vulnerable to secular socioeconomic, healthcare, and exposure changes. It should not be described as equivalent to a randomized intervention or a controlled difference-in-differences analysis. No verified instrumental-variable analysis was used to infer a causal effect in this review.

### 8.4. Pollutant Mixtures and Correlated Exposures

Modern mixture methods address different questions. Weighted quantile sum regression estimates a weighted index under a common-direction constraint; training and validation are needed, and correlated components can exchange weight [35]. Bayesian kernel machine regression can examine nonlinear joint associations and interactions, but its results depend on the data support, prior specification, and adequate sample size [36]. Quantile g-computation estimates a specified joint quantile shift and can accommodate components with opposing directions [37]. None of these approaches repairs unmeasured confounding, poor assessment of exposure, or a lack of independent variation between co-emitted pollutants. Component weights or inclusion probabilities should not be interpreted as independently identified toxic effects. Prespecified source-oriented contrasts, calibration data, correlation diagnostics and sensitivity analyses should accompany mixture models.

## 9. Public Health and Clinical Implications

The World Health Organization air-quality guidelines provide a health-protective benchmark and support continued source reduction even where legal air-quality standards are met [1]. Traffic-emission controls, clean public transport, industrial emission controls, clean household-energy transitions, land-use planning that reduces exposure near schools and childcare settings, and reliable monitoring networks can reduce exposure across populations.

Clinical counselling should be proportionate and non-stigmatizing. Air pollution should not be presented as the cause of an individual child’s ASD, ADHD, learning difficulty, or developmental delay. When families express concern, clinicians can acknowledge population-level associations, assess established developmental and medical risk factors, provide evidence-based surveillance, and refer for evaluation or support when indicated. Environmental counselling complements rather than replaces standard prenatal care, developmental screening, nutrition support, smoking cessation, and social services.

During severe pollution episodes, practical measures such as following local air-quality advisories, avoiding strenuous outdoor activity near heavy traffic, and reducing indoor combustion may reduce exposure. Such measures should not shift responsibility from emitters and public systems to families or imply that household filtration can offset persistent community emissions.

## 10. Future Research Priorities

Future studies should expand prospective birth-cohort infrastructure in highly exposed regions, use locally validated developmental assessments, and harmonize pregnancy dating, residential mobility, indoor sources, co-exposures, and clinical outcomes. Repeated personal monitoring, source apportionment and component measurements should complement residential PM_2.5_ and NO_2_ estimates. Pollution, noise, green space, and deprivation should be assessed at compatible spatial and temporal scales, with separate total-effect and joint-exposure questions specified before analysis. Larger samples do not compensate for systematically mismeasured exposure or omitted contextual determinants.

Longitudinal follow-up should distinguish transient variation in early test scores from stable functional impairment. Nested mechanistic studies can integrate placental measures, inflammatory and oxidative-stress markers, neonatal neuroimaging, and later developmental outcomes. Their value depends on pre-specified hypotheses, compatible timing, and careful interpretation of biomarkers as potential intermediates rather than causal proof.

### Strengthening Policy and Family-Based Designs

Natural experiments involving emission controls, clean-fuel transitions, low-emission zones, or changes in traffic can complement conventional cohorts. Difference-in-differences requires credible parallel pre-intervention trends and assessment of contemporaneous policies; interrupted time-series analyses require sufficient pre- and post-intervention observations. Within-family comparisons need meaningful exposure differences between pregnancies, and instrumental-variable analyses require a defensible exclusion restriction and adequate instrument strength. Triangulation across designs with different biases is more informative than relying on a single adjusted coefficient. Policy evaluations should document exposure reductions and clinically meaningful developmental outcomes.

## 11. Conclusions

Selected cohort evidence links prenatal and early-childhood ambient air pollution with small differences in some cognitive, language, psychomotor, behavioural, and neuroimaging outcomes, alongside substantial null findings. Interpretation remains limited by heterogeneous source mixtures, correlated windows, imperfect exposure measurement, incomplete co-exposure control, selection, and outcome ascertainment. Perinatal conditioning further separates conditional associations from total prenatal effects. Mechanistic observations and emission-reduction comparisons are informative but do not establish a single causal pathway or pollutant-specific effect. Reducing ambient and household pollution remains a prudent public-health objective. The evidence does not establish pollution as the sole or determinative cause of ASD, ADHD, or an individual child’s developmental profile. Geographically inclusive cohorts, better exposure validation and complementary causal designs are needed.

## Data Availability

No new data were created or analyzed in this study. Data sharing is not applicable to this article.
